# High Resolution Remote Sensing of Densely Urbanised Regions: a Case Study of Hong Kong

**DOI:** 10.3390/s90604695

**Published:** 2009-04-29

**Authors:** Janet E. Nichol, Man Sing Wong

**Affiliations:** Department of Land Surveying and Geo-Informatics, Hong Kong Polytechnic University, Hong Kong, China

**Keywords:** thermal remote sensing, urban heat island, aerosols, MODIS, PM_2.5_

## Abstract

Data on the urban environment such as climate or air quality is usually collected at a few point monitoring stations distributed over a city. However, the synoptic viewpoint of satellites where a whole city is visible on a single image permits the collection of spatially comprehensive data at city-wide scale. In spite of rapid developments in remote sensing systems, deficiencies in image resolution and algorithm development still exist for applications such as air quality monitoring and urban heat island analysis. This paper describes state-of-the-art techniques for enhancing and maximising the spatial detail available from satellite images, and demonstrates their applications to the densely urbanised environment of Hong Kong. An Emissivity Modulation technique for spatial enhancement of thermal satellite images permits modelling of urban microclimate in combination with other urban structural parameters at local scale. For air quality monitoring, a Minimum Reflectance Technique (MRT) has been developed for MODIS 500 m images. The techniques described can promote the routine utilization of remotely sensed images for environmental monitoring in cities of the 21^st^ century.

## 1. Introduction

**Figure 1.**
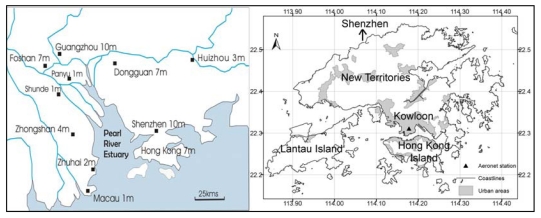
Urban areas of Hong Kong, and location adjacent to the large industrial cities of the Pearl River Delta with populations given in millions.

The synoptic view of cities afforded by remote sensing platforms confers great potential for data collection over urban areas although in reality urban areas have been the most challenging to remote sensing for a number of reasons. Urban areas are particularly challenging due to their high spatial and spectral variability coupled with the irregular size, shape and orientation of objects. Further constraints are imposed by limitations of the spatial and spectral resolution of the sensing systems. This paper demonstrates recent research in the fields of thermal remote sensing of urban microclimate, and satellite monitoring of air quality, which improves and enhances the spatial resolution of satellite image products for compatibility with intra-urban applications. Hong Kong (Figure 1) is used as a case study due to its uniquely high density urban environment, intense urban heat island (UHI) and deteriorating air and environmental quality over the last decade.

## 2. Measuring the Urban Heat Island

The last decade has seen urban temperatures in Hong Kong increasing by 0.6 °C, compared with a global increase of only 0.2 °C. Since there is no climate station in a densely urbanised location this may even be on the low side. Rising temperatures in tropical and sub-tropical cities mean that planners now need to consider adverse health effects such as respiratory problems due to increase in ground level ozone, and heat stress, and heat-induced mortality in their planning strategies. Measures include the influence of street layout, building geometry and surface materials. Due to the low resolution of the current thermal satellite sensors Landsat TM (120 m), Landsat ETM+ (60 m) and ASTER (90 m), there are no spatially detailed climatic data covering the whole city. Furthermore, the scan line corrector problem experienced by Landsat ETM+ since 2003, the lack of a thermal infra-red band in the Landsat Data Continuity Mission and the potentially long development period for NASA's newly announced Hyperspectral Infrared Imager [1], means that the highest resolution obtainable available from thermal satellite sensors in the foreseeable future is 90 m from ASTER.

**Figure 2.**
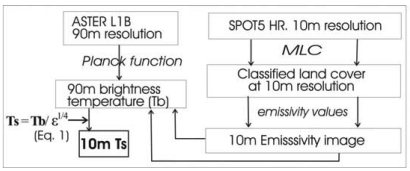
The Emissivity Modulation procedure: an emissivity image from classification of a SPOT 5 image with 10 m resolution is combined with a 90 m ASTER image of brightness temperature. Emissivity values are derived from the MODIS emissivity library.

A technique for enhancing the spatial resolution of thermal images, while simultaneously correcting the image derived temperatures for emissivity differences of surface materials is demonstrated [2]. The method combines a classified image derived from a higher resolution visible wavelength sensor, and a set of externally derived emissivity values with a lower resolution thermal image. It effectively ratios the visible and thermal datasets in the emissivity correction equation in a procedure derived from the Bolzmann's law (Equation 1, Figure 2).

**Figure 3.**
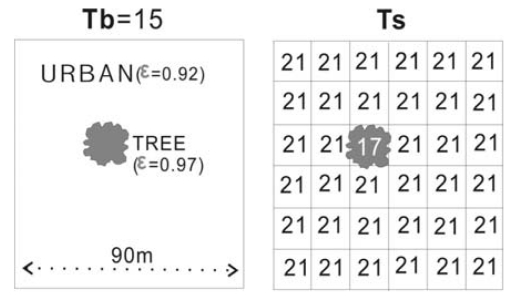
The effect of pixel block membership on fragmented land cover types in Emissivity Modulation.

This has the effect of simultaneously correcting the image-derived ‘Brightness Temperature’ (T_b_) to the actual Kinetic Temperature of the surface (T_s_), while enhancing the spatial resolution of the thermal data (Figure 3).

A tree canopy in a dominantly urban pixel block will have the same high T_b_ value (ie. 15 °C) as urban before emissivity modulation. This erroneously high brightness temperature for the tree will not be corrected by the EM process, but the temperature of the tree pixel will be further increased, though to a lesser extent than that of urban pixels.

### 2.1. Image and Field Data

The image used for the study was a night-time scene of Hong Kong obtained at 10:41 p.m. local time on a clear night on 31 Jan., 2007. Band 13 (10.25-10.95 μm) of ASTER's five thermal bands was selected for processing since bands 10 and 14, being close to the edge of the atmospheric window are most affected by atmospheric effects [3] and the sensitivity of band 12 is said to be decreasing [4]. The image was obtained as Level 1B radiance-at-sensor data, uncorrected for emissivity or atmospheric effects. The noise-equivalent temperature difference (NEΔT) of the ASTER thermal bands is given as 0.3 °C. The present study uses a night-time ASTER level 1B image at 90 m resolution covering both urban and rural areas of Hong Kong. It inputs this, along with a classified SPOT5 image at 10 m resolution, to the emissivity correction equation (Figure 2). The Emissivity Modulated (EM) image is verified using ‘*in situ*’ surface temperatures collected at the image time.

Eighteen paired surface and air temperature (T_a_) measurements were taken in both urban and rural areas across Kowloon and the New Territories, within one and a half hours of the image time, 10:41 p.m., with T_a_ offset by 1 m from the ground surface. The surfaces measured included homogeneous areas of asphalt, concrete, grass and water. Testo 720 portable digital thermometers with air and contact surface probes, having a stated accuracy of 0.2 °C were used, and each measurement represented the average of three readings. Before fieldwork, all sensors were placed in the same room with an Omega CL7304A calibrator to measure the same temperature for 24 hours, and a certified mercury thermometer NIST SRM943 was used as a standard reference. Temperature outputs of all thermometers were then normalized according to the correlation between each sensor and the reference thermometer. All field measured air temperatures were normalized to the image time by reference to hourly air temperature data from the Hong Kong Observatory located near the centre of the urban area. Atmospheric correction was carried out by adjusting the image T_s_ values to those of reference Sea Surface Temperatures (SST) recorded at the image time.

### 2.2. Validation of 10 m Emissivity Modulated Image

Both of the T_s_ images (with pixel sizes of 10 m and 90 m respectively) appear more accurate when compared with field measured T_s_ than the T_b_ image, but the EM image values are closest to the field data, with the lowest Mean Absolute Difference (MAD) of only 1.0 °C, and for points falling within the urban area, an MAD of only 0.5 °C. The EM image values were also the highest correlated with field T_s_ (r^2^ = 0.71), compared with r^2^ = 0.58 for the 90 m T_s_ image (Table 1).

**Figure 4.**
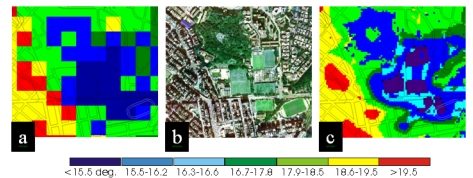
Image extract showing a 0.5 km^2^ area at the east edge of the heat island core of Kowloon. Temperatures in °C. (a) 90 m resolution emissivity corrected T_s_ image, (b) colour aerial photograph and, (c) Emissivity modulated T_s_ image at 10 m resolution.

The close correspondence of the EM image and actual land cover is illustrated in Figure 4, which shows micro-scale temperature patterns within a 0.5 km^2^ area. The extract is located on the east edge of the heat island core, where the old, high density commercial shopping district gives way to leafy low rise residential areas interspersed with parks and sports pitches (Figure 4b). Not only is the land cover boundary more distinct on the EM image (Figure 4c), but individual tree clusters and large buildings shown on the air photo (Figure 4b) can be identified by their temperatures at 10 m resolution (Figure 4c) whereas on the 90 m T_s_ image (Figure 4a) they are below the image resolution.

For urban heat island analysis based on air temperature, the high correlation (r^2^ = 0.66) in the field between surface and air temperatures at the image time suggests that image T_s_ would be highly correlated with T_a_. This fact is supported, with r^2^ = 0.72 for the EM image, which is substantially higher than for the 90 m T_s_ image with r^2^ = 0.56 (Table 1). This is surprising, given the recognised larger scale variability of air temperature, which was also noted on a mobile air temperature traverse conducted at the time. This may be explained by a greater mixed pixel problem in the image classification at 90 m resolution, leading to erroneous T_s_ values at 90 m resolution (for comparison with T_a_) and further supports the EM method.

### 2.3. Application of ‘High’ Resolution Surface Temperatures in Urban Heat Island Analysis

The EM method is not only able to increase accuracy in the representation of surface temperatures, but is also better able to indicate the strong influence of land cover type on the UHI, by showing greater temperature variation between classes and less variation within classes. This accords with Oke's [5] Physical Structure model, which asserts that the urban canopy layer heat island (ie. at street level within urban canyons) is governed more by the immediate site character, especially building geometry and materials, than by thermally modified air from upwind areas. In the Hong Kong context, this observation is directly applicable to current planning strategies as the older, medium rise, mixed commercial areas (Figure 4b left side), all coincide with heat island maxima, and should undergo redevelopment at lower density, using less absorptive materials, in order to mitigate increasingly oppressive temperatures. These areas are clearly identified as a compact and homogeneous region on the EM image (Figure 4c left side), but the boundaries are less precise on the 90 m T_s_ image (Figure 4a).

## 3. Air Quality Monitoring

Air quality in Hong Kong has deteriorated over the last decade, and the Hong Kong PolyU AERONET station shows aerosol levels to be high, compared with other urban stations worldwide, with, for example a mean AOT of 0.69 for the 440 nm band, compared with 0.57 for Beijing, 0.55 for Singapore, 0.22 for Rome, and 0.24 for the Goddard Space Flight Center. It is likely that the largest proportion of Hong Kong's pollution originates from adjacent rapidly industrialising areas of the Chinese mainland, but with only 14 air quality stations, the occurrence and intensity of trans-boundary air pollution is difficult to establish. In Hong Kong, air quality modelling by the Environmental Protection Department (EPD) suffers from the distant location and uncertainty of the data sources outside Hong Kong, making model output at resolutions higher than 1.5 km meaningless. Even at this coarse resolution very little variation in air quality over the 1,060 km^2^ of Hong Kong's territory is evident, although data from the 14 ground stations suggests substantial spatial variation [6].

There is currently no reliable method for the monitoring of air quality at detailed levels over urban areas using remote sensing, due to the following reasons. Methodologies by Tanré *et al.* [7], Sifakis and Deschamps [8], Kaufman and Tanré [9], Hsu *et al.* [10] do not provide consistent results over spatially complex regions due to inadequate spatial and temporal resolution, combined with a lack of suitable algorithms. According to Li *et al.* [11], aerosols over a 50 km^2^ domain do not vary much, except over regions near major emission sources, and most previous remote sensing studies have not addressed variability in air quality at fine resolution. Thus the MODerate Resolution Imaging Spectroradiometer (MODIS) standard Aerosol products (known as collection 4 and collection 5) are at the coarse resolution of 10 km. Furthermore, it was devised for MODIS wavebands to measure the transparency of the atmosphere over areas of dense vegetation which are dark in blue and red bands. Chu *et al.* [12] revealed that the MODIS collection 4 algorithm has a positive bias when compared with the AErosol RObotic NETwork (AERONET) sunphotometer data [13]. Recently, Levy *et al.* [14] devised collection 5, which modifies the surface reflectance determination by considering a band correlation based on vegetation index (NDVI_SWIR_) and the scattering angle. Although significant improvements in collection 5 algorithm was shown both in terms of accuracy and continuity of aerosol retrieval [11,15], the southern China region especially Hong Kong and the PRD region has been identified as having a large error in AOT retrieval [9]. The only ‘high’ resolution aerosol study, by Li *et al.* [16], who applied the MODIS collection 4 algorithm at the resolution of 1 km to retrieve AOT over Hong Kong, was limited to dark vegetated areas and the results were validated only between October and December 2003 using handheld sunphotometers.

To overcome the two major limitations of collection 4 and collection 5 data for urban scale study in Hong Kong, namely bright urban surfaces such as urban areas, and low spatial resolution, a new aerosol retrieval algorithm using the MODIS 500 m resolution bands was developed. The rationale is to estimate the aerosol reflectances by decomposing the Top-Of-Atmosphere (TOA) reflectance from surface reflectance and Rayleigh path reflectance. For the determination of surface reflectances, a Minimum Reflectance Technique (MRT) is used, whereby MRT images which are a composite of the lowest value pixels over a number of different dates, are computed. The minimum reflectance technique (MRT) was developed for TOMS [17] and GOME [18] data at coarse resolution (> 1 degree), and in view of the coarse resolution the accuracy of AOT that was achieved (within 30% of AERONET ground measurements) could be considered good.

### 3.1. Data Used

A whole year (2007) of TERRA/MODIS level 1B calibrated reflectance images (MOD02HKM) at 500 m resolution, and MODIS level 2 aerosol products (MOD04) at 10 km resolution was collected. Validation of the AOT retrieved from our new methodology was carried out by comparison with the Hong Kong AERONET station. In order to validate the surface reflectance estimated from our MRT method, the MODIS surface reflectance products (MOD09 8-days composite surface reflectance images) were also acquired from NASA Goddard Earth Science Distributed Active Archive Center (DAAC) for the year 2007. The MOD09 surface reflectances images are corrected for the aerosols, gasses and water vapour using the inputs of MODIS atmospheric data. They are then validated with 150 AERONET stations and are considered acceptable if the data error lies within ± 0.005 + 5% [19].

### 3.2. Methodology of Minimum Reflectance Technique

The MRT method obtains surface reflectance by creating an image surface reflectance composite, which is comprised of pixels having the lowest value from many images over a time period. To minimize seasonal land cover changes, seasonal minimum reflectance images were derived based on at least thirty clear-sky images for each of four seasons. Then, the second minimum reflectance values (rather than the actual minimum) were retrieved in order to avoid abnormal low reflectance such as noise or shadow. In addition, clouds were removed by thresholding the thermal waveband, which is essential in cloud-prone areas like Hong Kong. Only nadir images with satellite viewing angle < 35° were considered in order to minimize the angular effects caused by bidirectional reflectivity in heterogeneous areas.

### 3.3. Aerosol Retrieval

Aerosol reflectance was calculated for different aerosol types specific to our Hong Kong study area, using local AERONET data, sun-viewing geometries and relative humidity (RH), by construction of a LUT using the SBDART code [20]. A few aerosol models from the Optical Properties of Aerosols and clouds (OPAC) [21] database were used. These include i. continental clean (CC), ii. continental average (CA), iii. continental pollutant (CP), iv. desert dust (DD), v. maritime clean (MC), vi. marine pollutant (MP) models. These 6 aerosol models with 9 solar zenith angles (0°∼80°, Δ = 10°), 17 view zenith angles (0°∼80°, Δ = 5°), 18 relative sun/satellite azimuth angles (0°∼170°, Δ = 10°), 8 RH values (RH = 0%, 50%, 70%, 80%, 90%, 95%, 98%, and 99%) were considered in the LUT. These parameters gave a possible 132,192 combinations for 3 bands (470, 550, 660 nm), for computing the hypothetical AOT. An optimal spectral shape-fitting technique was executed to select the aerosol model with the smallest systematic errors [9,22,23] (Equation 1).

(1) $$x^{2}=\frac{1}{n}\sum_{i=1}^{n}{\left(\frac{\rho_{\text{Aer}}^{m}(\lambda_{i})-\rho_{\text{Aer}}^{a}(\lambda_{i})}{\rho_{\text{Aer}}^{m}(\lambda_{i})}\right)}^{2}$$

The error term of *x*^2^ is described as the residual of the measured aerosol reflectances $\rho_{\text{Aer}}^{m}(\lambda_{i})$ from MODIS and modeled aerosol reflectances $\rho_{\text{Aer}}^{a}(\lambda_{i})$ from aerosol models. The minimum residual of *x*^2^ is selected from the six aerosol types for each pixel. Thus, the appropriate aerosol type is selected and the corresponding AOT values are then derived for each pixel.

### 3.4. Validation of the MRT Surface Reflectance

Strong correlations between MRT images and MOD09 were observed in the fall and winter seasons (r^2^ > 0.8), while moderate correlations were noted in the spring season (r^2^ > 0.6). The differences in surface reflectances (y-intercepts of the slopes) were less than or equal to 0.01 (1% reflectance unit) for the 550 and 660nm wavelengths while the differences were greater for the shorter 470nm wavelength (∼0.02-0.03) (2-3% reflectance units). This gives a potential AOT error of 0.2-0.3 due to the error in surface reflectance, if absolute accuracy of the MOD09 images is assumed. However such accuracy cannot be assumed due to the errors of atmospheric correction in the MOD09 products [19]. Thus we estimate the uncertainty of our AOT values induced by the error of MRT surface reflectances to be in the range of 0 to 0.3, and to be greater and significant in the shorter wavelength (470 nm) but minimal at longer wavelengths (550, 660 nm).

### 3.5. Validation Using AERONET Measurements

**Figure 5.**
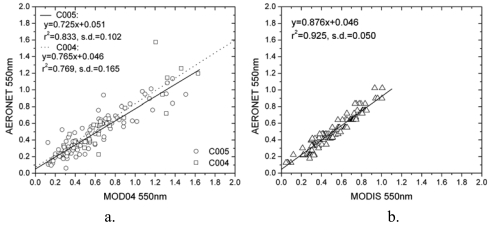
(a) MOD04 collection 4 and 5 AOT compared with AERONET measurements; (b) MODIS 500 m AOT compared with AERONET measurements.

Good agreements are shown between the MODIS 500 m AOT at 550 nm derived from our methodology and the AERONET (Figure 5b) with a linear-fitting correlation coefficient (r^2^) of 0.93, which is higher than AOT from MOD04 collection 5 (r^2^ = 0.83) and MOD04 collection 4 (r^2^ = 0.77) (Figure 5a). The slope of the linear-fitting equation is close to unity, and suggests a slight underestimation in AOT of ca. ∼0.046.

**Figure 6.**
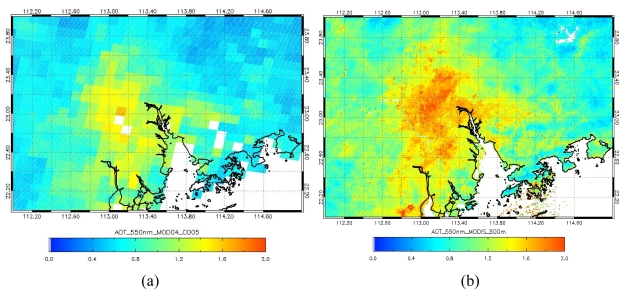
AOT images of Hong Kong and Pearl River Delta region on 20 Oct, 2007; (a) MODIS MOD04 standard product at 10 km resolution, and (b) MRT image at 500 m resolution.

Most importantly, the method described here can retrieve AOT images at a spatial resolution high enough to show detailed patterns of AOT over complex terrain with neighbouring bright urban and dark rural vegetated areas (Figure 6b). This improvement is significant for a topographically complex area like Hong Kong and the neighbouring mega-city region of the Pearl River Delta (PRD).

### 3.6. Application of the 500 *m* AOT Images to Aerosol Monitoring

**Figure 7.**
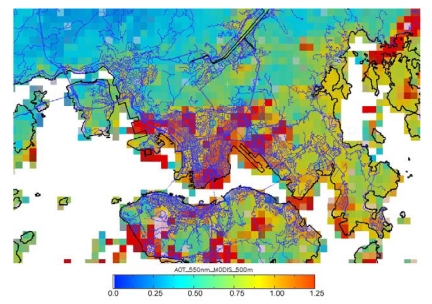
AOT image of Hong Kong on 20 Oct, 2007, derived from MRT technique at 500 m resolution.

The AOT distribution over Hong Kong and the PRD region on 20 Oct, 2007 retrieved using MODIS collection 5 AOT image at 10 km resolution (Figure 6a) is visually and spatially inferior to the 500 m AOT image (Figure 6b). The AOT on this date was relatively high with a range of ∼0.6 in rural areas to ∼1.6 in urban areas. However, only the 500 m MRT image (Figure 6b) indicates that the northern part of Hong Kong especially near the Chinese city of Shenzhen suffers from cross-boundary pollutants which are emitted from industries in the PRD region, and here AOT values above 1.0 are observed. The figure shows severe air pollution in these areas due to the emissions of the many industries and power plants located here. Figure 7 shows the effectiveness of the 500 m AOT image for aerosol monitoring in urban areas, and that overlay with the road network can highlight pollution levels corresponding to specific city districts.

The MRT method for aerosol retrieval, coupled with comprehensive LUTs which consider many different aerosol models, sun-satellite geometries and RH conditions specific to the local Hong Kong and PRD region appears to offer a method for accurate estimation of aerosols over both urban and vegetated areas at a high level of detail.

## 4. Monitoring of Fine Particulates

### 4.1. Study Description

Fine particles in the atmosphere have recently been recognized as the most serious pollutant for their damage to the circulatory and respiratory systems [24]. Hong Kong has 14 air quality stations measuring hourly pollutant gases and particulates, but only five of them are equipped to monitor PM_2.5_. These indicate that the annual average PM_2.5_ concentrations for the years 2005, 2006 and 2007 are high, at 60, 56, 53 μg/m^3^ respectively, and exceed the US EPD 24-hour air quality standards which were upgraded from 65 to 35 μg/m^3^ in 2006. In order to investigate the retrieval of fine aerosols from satellite image data, a whole year (2007) of AOT images was extracted for pixels corresponding to the five PM_2.5_ stations. This was done using regression against the hourly-collected PM_2.5_ data at the 5 ground stations were selected to be within 30 minutes of the MODIS overpass time. Both the 10 km AOT MOD04 product, and the AOT at 500 m resolution derived from the MRT method described above were evaluated.

### 4.2. Findings and Recommendations for Intra-Urban Monitoring of Fine Particulates

Kumar *et al.* [25] observed large variations in PM_2.5_ concentrations within 10 × 10 km pixels, from measurements at 113 ground locations in Delhi. This is commensurate with the low to moderate correlations obtained by most previous studies using coarse (10 km or coarser) resolution AOT datasets [26-29], which generally fall short of the ca. 80% reliability required for an operational model, unless some parameters are controlled. This is the first study to examine relationships between satellite-derived AOT and ‘*in-situ*’ PM_2.5_ concentration, at different resolutions including local (intra-urban) scale.

**Figure 8.**
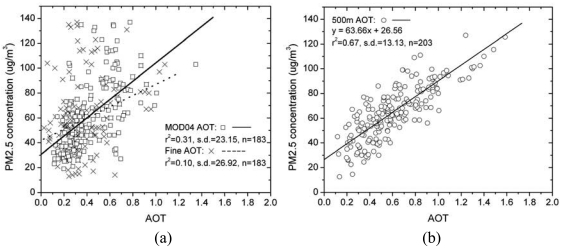
PM_2.5_ concentrations at ground stations compared with MODIS AOT at (a) 10 km resolution and (b) 500 m resolution.

We found higher correlations between AOT and PM_2.5_ at 500 m than at 10 km resolution (r^2^=0.67 and 0.31, respectively; Figure 8). The AOT images derived at 500 m are able explain 67% of the variance in PM_2.5_. Assuming the AOT images are accurate, the remaining 33% of the variance not explained may be due to dimensional and scale discrepancies between ground level PM_2.5_ distribution and 500 m pixels, as well as other parameters unrelated to AOT such as atmospheric pressure, stability within the atmospheric boundary layer, and humidity. This preliminary study demonstrates great potential for the routine use of satellite-derived AOT as a surrogate for detailed PM_2.5_ distributions at ground level. The study assumed a stable planetary boundary layer (PBL) over Hong Kong, with most aerosols concentrated in a well-mixed boundary layer near the ground. For refinement of the model, Lidar instruments deployed in different areas would be useful to evaluate the influence of aerosol vertical distribution and PBL heights. As more data become available, different regression models constructed for humid and non-humid seasons and different PBL heights should prove more robust, and enable the Environmental Protection Departments in Hong Kong and the industrialised Pearl River Delta to derive fine particle distributions over the region at detailed level from daily satellite images.

## 5. Conclusions

All three studies described above were able to obtain higher accuracy compared with ground data, using fine spatial resolution satellite image products, although the variables being measured generally exhibit low spatial variability. However, within a densely-built urban environment such as Hong Kong, as well as the compact, high rise mega-cities predicted for mainland China, air temperature and air quality may be expected to show greater spatial variability due to intensive juxtaposition of land uses. In view of the lack of high spatial resolution sensors planned for the near and medium-term future, the image processing methodologies presented here will enable more accurate and relevant urban environmental modelling.

## Figures and Tables

**Figure 9.**
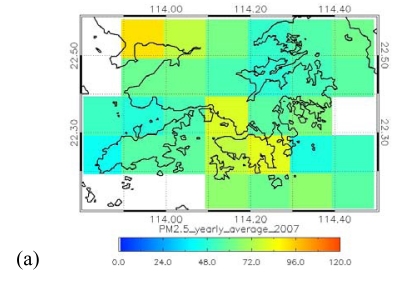

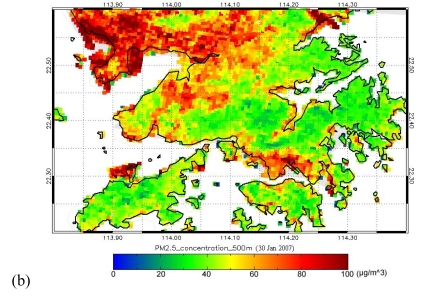
Distribution of yearly average PM_2.5_ (μg/m^3^) over Hong Kong in 2007, from (a) MODIS collection 5 data at 10 km, and (b) modeled PM_2.5_ at 500 m resolution on 30 Jan., 2007. Current US EPA standard is 35 μg/m^3^ over a 24-hour period.

**Table 1.** Comparison of ‘*in situ*’ measured surface and air temperatures with image data. MAD stands for Mean Absolute Difference.

**Field data**	**Image processing method**	**r^2^**	**MAD**

Surface field	T_b_ 90 m	0.45	2.2
Surface field	T_s_ 90 m	0.58	1.4
Surface field	T_s_ 10 m (EM method)	0.71	1.0
Air field	Surface Field	0.66	1.4*
Air field	T_b_ 90 m	0.31	
Air field	T_s_ 90 m	0.56	
Air field	T_s_ 10 m (EM method)	0.72	

* Mean difference
